# Maryland State Dental Association

**Published:** 1892-07

**Authors:** 


AMERICAN JOURNAL

—OF—

DENTAL SCIENCE.

VOL. XXVI. BALTIMORE, JULY, 1892. NO. 3.

ARTICLE I.—ORIGINAL.

### MARYLAND STATE DENTAL ASSOCIATION.

The annual session was held May 9 and 10, 1892, in St. James' Hotel, Baltimore; Cyrus M. Gingrich, D.D.S., President, in the chair; W. W. Dunbracco, D.D.S,, Secretary.

(Continued from page 61.)

### REPORT OF COMMITTEE ON OPERATIVE DENTISTRY.

BY A. N. SWEENEY, D. D. S.

Since the last meeting of this Association the publishers of dental literature have not been called upon to chronicle any new and startling discovery. No bold and brilliant operation has been introduced to furnish the topic for general discussion, the *perfect* filling material has not been discovered, neither has any method to obviate all necessity for fillings, through the prevention of caries, been devised.

Upon more careful consideration, it would seem to be a rather humiliating acknowledgment to state that a number of months should roll around without the presentation of *something* worthy, at least of passing consideration, either in the line of new remedies, appliances or methods, or in the detection of some old errors hitherto masquerading in the guise of truth, and it is the hope of being able to call attention, in a very general way, to some item which may furnish food for thought and stimulate useful discussion, which must be offered in justification of this paper.

The field of therapeutics is a wide one, in which many enthusiastic workers are constantly engaged, and we see the fruit of their labors brought forth, from time to time, in the form of new preparations, or old ones under new and improved forms. Some of these are of especial interest to the dentist, and though very many fall far short of realizing the hopes of the introducers, this does not serve to deter investigation; but, on the contrary, the repeated failures seem only to stimulate to greater zeal and increased activity.

That class of preparations known as germicides continues to absorb a great amount of attention, and though some one has lately complained that we are at present suffering from the effects of a "fever" or "boom" in this direction, which must soon subside, no one can close his eyes to the magnificent achievements which have been the reward of investigation of this one line of remedies, for in no other department of scientific research, perhaps, has the success been greater, and in none is investigation pushed with greater enthusiasm. What the perfect germicide of the future will be, it would be unwise to predict. That it has not yet been determined upon is evinced by the fact that the search still goes on. Indications seem to point to the conclusion that the bi-chloride of mercury does not fulfill all expectations. Those of you who attended the last meeting of this body had the pleasure of witnessing a demonstration of the diffusive properties of pyoktannin and listening to an interesting description of its various virtues by the late, lamented Dr. W H. Atkinson; and yet the interval which has elapsed since then seems to have con·igned pyoktannin to a place among the long list of failures.

A paper lately read by Dr. H. A. Kelly of the Johns Hopkins University places the long familiar permanganate of potash in the very front rank of germicides, while carbolate of camphor is also a candidate for first honors in that class.

As a boon to both dentist and patient, who have alike suffered long from the offensive odor of iodoform, it may be stated that aristol seems to possess all its virtues without its one glaring vice.

Some progress has been made in the direction of the long-felt want of a practical, safe and efficient obtundent of sensitive dentine. The most recent efforts to supply this need seem to have been directed toward the attainment of this end through the application of such directly opposite agencies as heat and cold, and while there is nothing new in principle with either method, the means of application have lately been much improved. Hitherto any apparatus designed for the application of intense cold has been more or less complicated, expensive and variously objectionable, but we now have provided in the little glass tubes of ethyl chloride something at once practical, convenient and cheap, which will be found effective in many cases, while the means of applying heat, either alone or in conjunction with some drugs, have also been much improved of late. A detailed description of a number of these appliances and their action will be found in a late issue of *Items of Interest.*

Some recently published reports would seem to indicate that the subject of the conservative treatment of the pulp was, just now, receiving rather more attention than it has appeared to have received for some few years, and though it is hardly to be desired that we should see a revival of the attempts to amputate portions of this organ and preserve the remainder in a state of usefulness, such as were made some years ago, it is a subject worthy of attention and one which should receive always due consideration. To devitalize promiscuously every pulp which may offer, simply because there may be more or less exposure, is clearly bad practice, yet it is probably the practice of many.

The treatment of peri-cemental inflammation and alveolar abscess naturally brings us back to the consideration of germicides; but while the chemists are busy at work trying to produce the infallible preparation, which shall prove to be "sure death" to all germ life, the busy, every day dentist, who, as a rule, is not a brilliant success as a chemist, can rest tolerably easy in the knowledge of the fact that with a few of the old reliable preparations, which all have known since they were boys, backed by a fair amount of brains, and assisted with a good outfit of modern instruments, can, provided he will honestly and faithfully use these means, cure nearly all his abscesses. A member of this Association has lately been meeting with very gratifying success in the treatment of these cases by the use of listerine alone, which he supplies to his patient and directs him to use thoroughly and frequently; but as the gentleman is present, the details of his treatment had better be obtained from him in person, since it is not the writer's wish "to steal his thunder."

Of the various filling materials amalgam now attracts a great deal of attention. A vast amount of study and experiment have been devoted to this subject, with the result that many most excellent alloys have been compounded and are now obtainable. The senseless prejudice against this class of filling which existed up to a recent date has largely passed away, and it has come to be generally accepted that amalgam is not, *per se*, simply a cheap and inferior substitute for gold. Now that the falsely nurtured discredit which attached to the use of this material has disappeared, in addition to the regular manufacturers of the article, many dentists are earnestly engaged in turning it out under formula of their own, so that we may now be said to possess, in addition to the regulars, a class presenting a seeming anomaly, that is a class of professional people who are yet amateurs, amateur amalgam makers, and the result of their combined efforts is that the number of "brands" turned out almost passes computation. This is praiseworthy, however, and many excellent samples of alloy have been produced by the amateurs. One of the very latest candidates for favor has brought out what is termed an aluminum amalgam, and it is possible that the so-called "metal of the future" may partially substantiate its claims through the excellence of the alloys of which it may form a component part. But in the list of alloys, white and colored, there seems to be one "black sheep," of which the worst was not said when it was pronounced to be "a nigger." Copper amalgam has not fulfilled the wonderful promises made for it. Many careful members of the profession were very shy of it from the outset, and many of those who rushed headlong into its use, in the belief that they possessed in it "the one thing needful" (in an amalgam) have had ample time to repent at their leisure.

Among other plastics, the oxy-phosphate of copper is now attracting attention, and it is to be hoped that the metal may, in that form, atone for its shortcomings as an alloy.

About June last Dr. Timme, of New York, visited this city and introduced his process of making inlays, presenting in it a simple and practical method, fully within the reach of all, of employing this useful method of filling conspicuous cavities. It may be noted also right here that a number of ingeniously designed rubber-dam clamps intended to facilitate the filling of labial cavities have been lately put upon the market, showing that this troublesome class of cases has been receiving renewed attention.

The subject of gold filling is one about which it might seem best to remain silent, for the amount of writing already done and the discussion indulged in about it have been simply immense. To the outside observer it might seem to be the most incomprehensible thing about the dental profession that the matter of how to fill teeth with gold was not definitely settled long ago, so that discussion might have had time to cease. Gold has been used to fill teeth for many years, and gold is a simple substance, unlike an alloy. Where, then the cause for so much contention? Almost all dentists fill with gold; what proportion of them do so with any near approach to true success?

Your last report dealt at considerable length with this subject, and treated it in so plain and practical a manner as to obviate all need for details at this time. While disclaiming all desire to detract from the merits of non-cohesive gold, the paper took strong grounds in favor of the cohesive preparations. Undoubtedly, from the standpoint of a majority of those in practice to-day, these views are correct. The discussion which followed the reading of the report developed the usual differences of opinion, for while there were those who were ready to champion the cause of non-cohesive gold, the advocates of the cohesive were well represented, and one of their partisans so thoroughly felt "the courage of his convictions" as to declare that "any cavity that can be filled at all with non-cohesive gold can be *better* filled with cohesive." Unfortunately, of the comparatively few members of the profession who are recognized as holding the position of teachers to their brethren, by virtue of their gifts as writers and public speakers, many have fallen into the habit of investing their individual ideas with too great a semblance of authority. This is to be regretted, as the willing disciple is often perplexed when confronted with some point on which the "doctors disagree." Now had our old friend contented himself with a personal application of his views, and said that he, himself, could fill any given cavity better with one form of gold than with another, no exception could be taken to his remarks. For some reason, probably the result of early training in many cases, the preference for one or the other of the preparations of gold known as cohesive and non-cohesive amounts to a passion with very many dentists, which causes them to stick to their particular idol with a tenacity of purpose which finds almost its only parallel in the attachment manifested to early inculcated religious or political faith : hence statements similar to the one above quoted, or the converse of it, are not infrequent. In a series of articles from the pen of Dr. Ottolengui, now running in the *Dental Cosmos*, under the head of "Methods of Filling Teeth," the author expresses himself as unable to discover any use for the non-cohesive form of gold whatever.

Now the truth of this matter would seem very easy of discovery. The requirements of a gold filling are not so wonderfully numerous or complex as to be with difficulty understood. Its main use is to fill up the space left vacant by loss of tissue, caused by disease, in such a manner as to arrest further waste, to restore function and comfort to the tooth, and to present a reasonably good appearance. Leaving large contours out of the question, since there should be none with regard to the preparation of gold used for such operations, any two simple fillings which will show equal durability, comfort and good appearance are equally good, without regard to the fact that one is made of cohesive gold and the other of non-cohesive.

Where results, then, are equal, what advantage can one preparation have over the other ? Simply this, that preparation of gold is best for a given case which can be successfully introduced at the minimum cost of strain upon the patient and fatigue of the operator. Results being equal, he is the better operator of two men who can select his preparations of gold so as best to suit his cases, and, on occasion, is able to use them all well. It may be said, therefore, with profit alike to the all-hand-pressure as to the all cohesive gold operator, Go learn the other method. Put the small end of your opera glasses to your eyes, and you will be surprised how the field of vision is expanded.

When the strictly cohesive gold operator admits the need of some form of sponge gold to start certain fillings, on account of its softness and easy adaptability, and yet deplores the tendency of such gold as Steurer's to crumble to pieces in handling, let him provide himself with some of Abbey's non-cohesive foil, and he will find that it too has "adaptability," while ordinary handling will not injure it in the least, and he can finish out his filling with cohesive gold from whatever point his judgment may lead him to consider best. Should he find difficulty in handling this gold at first, some good, old friend who practiced when his father was a boy will take pleasure in showing him how to manage the wedging. True, it has been charged that this particular brand of gold has some impurities about it which serve to render it so utterly non-cohesive, but Dr. Bonwill told you that he had gone through the works and examined very carefully, and that the charge is false. Let that be as it may, the gold will stand in the mouth, and that is the practical issue. On the other hand, to see a man laboring over a large filling, crowding in sheet after sheet of gold by hand pressure, and a great amount of it, in these days when equally good or better results can be attained with so much less waste of energy, is to experience feelings of pity, chiefly for the unfortunate patient.

The various forms of mallets now in use are likewise subjects of preference and prejudice among those who use them, and here again comes in the danger of allowing one's judgment to be warped by the latter influence. That useful old instrument, the automatic, has lately come in for a rather severe arraignment at the hands of a popular writer who states that he used it extensively until it was, one day, used on himself. Experience shows that individual patients vary greatly in their estimate of certain appliances. A highly nervous lady, who could never tolerate the automatic, was delighted the first time the electric mallet was used, and insisted on its use ever after, while a similarly nervous gentleman pronounced the electric mallet "the vilest thing" he had ever seen, and expressed great satisfaction when the automatic was substituted; so it may be readily seen that individual peculiarities of temperament play an important part in determining the selection of appliances to be used with different subjects.

It is the writer's opinion that a skillful operator should be familiar with the use of all the three forms of mallets, namely the hand, the automatic and some form of power mallet, and should be fully able to fill successfully entirely by hand pressure.

As there is a separate paper on crown and bridge-work, it is unnecessary and would even be discourteous to attempt anything more than a simple allusion to the subject here. Crowns, in their now numerous forms, are very generally adopted by the profession. They are a great boon, and perhaps the only caution which may be mentioned with regard to their use, apart from the well-known necessity for great care in their adjustment, is to avoid a too hasty recourse to such a radical method of treatment, in many cases where moderately extensive fillings would serve as good or, possibly, a better purpose.

Bridge-work is still regarded by a large number of practitioners with distrust. The cause of this is not far to seek, when it is disclosed that many base their opinions of this work upon the experience of persons who have attempted to wear improperly or unskillfully constructed appliances miscalled "bridges." Such as are doubtful of their ability to successfully cope with the difficulties of this work had far better leave it to other hands; and certain it is that if there be any field for what is commonly called "cheap dentistry" it is not discoverable in the direction of making this class of applicances, for all poorly and carelessly constructed bridges are exceedingly likely, not only to prove highly unsatisfactory to the wearers, but will serve to bring discredit upon this great achievement of modern dentistry.

But little has appeared in print for some time about the operation of implantation, though some items upon this subject, and the kindred one of replantation, are to be seen in some of the publications for the current month. Had time permitted, it would have afforded pleasure to have communicated with the eminent man whose name must ever be associated with this operation, and had from him the results of his later experience.

It is a matter of regret to the writer that a paper containing so much good, practical advice, and, in the main, so commendable as the last report presented to you by the committee on operative dentistry, should have advocated so strongly the practice of extracting the sixth year molars. These teeth are declared to be, in the present generation, but little more than temporary, and it is advised that, in nine cases out of ten, they had better be extracted.

Some adverse criticism was noted against this portion of the paper at the time it was read, but the discussion wandered into other channels, and the acceptance of the report, as a whole, caused a notice to appear subsequently in the *Dental Cosmos* to the effect that this Association endorsed, among other recommendations, the extraction of the sixth year molars.

Whatever may have been the individual views of the majority of those present at that time upon this subject, the writer feels called upon to protest, in no uncertain manner, against the continued adherence to this antiquated method of practice, and hopes that the Maryland Association will go on record as in accord with many of the bright and progressive spirits of the profession at large, who are at this moment speaking and writing in earnest protest against the continuance of this pernicious practice. You heard the charge in your president's address last evening that "we have done very little in Maryland to add to the material interests and elevation of the profession at large." In the name of reason, do not lay us open to the far graver charge that Maryland constitutes the last stronghold of barbarous and obsolete methods.

To assume that the usefulness of a set of teeth, as a whole, could, in the great majority of cases, be increased by the loss of the largest and strongest of its number, would not seem, on the face of it, to be in reasonable conformity with natural laws, no matter whether we recognize in these laws the workings of a Divine Will, evolution or transformism. If these teeth be so nearly worse than useless, and if it be true that they are so rapidly progressing, rather retrograding, toward the temporary type, where are the evidences of any effort of nature toward their suppression?

The popular idea that these teeth are essentially so much more frail than their neighbors is, at least, possibly open to question. True it is they are very frequently found at an early age suffering from extensive ravages of caries; but the ignorance and neglect of parents will often as nearly account for this state of affairs as will any real structural inferiority of the teeth, and in a great number of instances where they escape the critical period of early life, they will be found to compare very favorable with the other teeth. But whether more frail or not, there are now resources at our disposal which may be made available for their salvation, hence to condemn them to almost wholesale slaughter would not seem in keeping with the spirit of the present age.

For the correction of irregularities the removal of some teeth will often become necessary, yet even for *this* cause there is a decided, and apparently growing, opposition to the arbitary selection of the first molars for extraction. In order to demonstrate that the views here advanced are not merely individual, but that they are shared in by certain writers and thinkers in the profession whose opinions are entitled to respect, and that the only motive in bringing them forward is the hope of keeping the members of this Association "in touch" with the best modern thought of the profession, a few random quotations from recent publications will be offered in closing. Dr. Ottofy, in *Items of Interest*, March, 1892, says: "I think it bad practice to extract the first molars, when badly decayed, with the hope that the second and third will fill the space. I do not think, with the methods we now have of saving the first molar, that, generally, it need be extracted and we do not gain as much by that practice as we desire. The articulation becomes disturbed, both by the dropping of the teeth forward and backward, inward and outward. The second and third molars seldom articulate as properly if the first molars are absent as when they remain. Also, unless the second molar moves forward" (which it frequently does not) "we will lose an exceedingly large tooth, and one which is better adapted for mastication than any other by virtue of its location and the relation it bears to the fulcrum and the muscular power exerted by the jaws. We can exert more force at that point than at any other; hence, when possible" (to avoid it) "it is not good practice to extract that tooth with the hope of having some other tooth do as much good."

Dr. Ottolengui says in *Cosmos* for this month, speaking of badly decayed first molars, "So far from considering these teeth good subjects for extraction, I take unusual pains to save them."

In the report of a paper read by Dr. H. V. Jackson at the meeting of the American Dental Association in August last, published in the *International Journal* for the current month, occurs the following: "The extraction of teeth that is so common to prevent crowding should be discarded. If there be insufficient room for the eruption of the teeth, it is better to expand the arch, which is easily done by means of the crib. Correcting the position of inferior incisors will often correct the superior if done while they are erupting."

In *Items of Interest* for April 1802 a question is asked by some one as to whether the first molars or two of the bicuspids had better be extracted in order to correct an irregularity of the cuspids. The answer is given editorially that, all things considered, the bicuspids had better be taken. It is admitted that the first molars may be selected, should the querist much prefer it, but he is warned that such a course "is likely to result in more experience than satisfaction."

To mention the name of Dr. N. W. Kingsley, is to cite a universally acknowledged authority on irregularities of the teeth. In the *Dental Cosmos* for January, 1892, occurs the following very candid statement from him, in giving a description of a case which he had treated: "As a proper correction of this deformity required the incisors to be carried backward somewhat, and as there appeared to be no spaces in the arch which would permit of its contraction the first, or sixth-year, molar on each side was extracted.

"These particular teeth were chosen for extraction in preference to the first bicuspids (which in many cases of protruding incisors would be proper), because they offered more resistance to the correction of the malformation of that region than any others, and *that*, together with the apparent necessity for extracting one tooth on each side to provide for retraction, seemed ample justification for the removal of two perfectly sound molars." But in a foot-note at the bottom of the page he says, "I am convinced by a study of the case since the correction was completed that the extraction of those permanent teeth was unnecessary. I find now to my surprise, that although there then appeared no room to carry the incisors back without extracting two teeth, there are now ample spaces among the teeth on each side to permit of another tooth."

In the face of such testimony as to the doubtful results of extraction even for the purpose of treating pronounced irregularity, what must be thought of making it a general rule of practice where no marked irregularities present?

After having gotten some of the "experience" to which the editor of *Items* refers in the treatment of irregularities where the first molars had been extracted, to say nothing of witnessing the spectacle of a lovely society belle, one side of whose inferior maxilla was bent almost into the form of a letter V, from the ill-advised removal of a first lower molar, leaving the crown of the second molar tilted forward very much and almost half an inch short of articulating with the upper teeth, it is scarcely to be wondered at that the writer *does not* favor the general extraction of the sixth year molars.

After reading his paper Dr. Sweeney said: "I would say that, to my mind, any one who had the pleasure of being here this morning and listening to the address of Dr. Bonwill, even though, before this, he had some lingering preference for the extraction of the sixth year molars, must have been converted."

DISCUSSION.

Dr. David Genese.—While the report which you have just heard calls for some remarks it is difficult to attack. I will attack it at its weakest point, copper amalgam. In the year 1867 I put some copper amalgam in the first molar for a gentleman in England and about five years ago the filling was still good. The turning black of copper amalgam is caused by using an iron bar in filling the copper in, for this produces copper sulphate which quickly turns black on being exposed to acid.

Now the point of porcelain inlays. I have experimented with them and they failed every time. I find that the fillings instead of being porcelain are glass just covered with a light coloring. I advise the method of filling the matrix with wax, then you can make a true inlay. Now comes the most difficult part of all and that is cementing it into the tooth. You all know that a polished surface will not permit it. After waxing the surface of the inlay I make scratches through the wax and then put it in hydrofluoric acid. In this way I get a fairly rough surface.

Now about the conservation of the sixth-year molars. I have given much attention to this subject. I must say that my success in this city has come from conserving the sixth year molars.

Dr. E. P. Keech.—I want to commend the paper and to express my thanks to Dr. Sweeney for what I consider an exceedingly well written paper on this subject. In regard to the sixth year molars I am very glad that Dr. Sweeney has taken the position he has. It has always been my practice to conserve the sixth year molar tooth for the reasons stated by Dr. Sweeney. Being the largest and strongest they are the most useful grinders of the teeth. In regard to the view that the second and third molars move forward and take the place of the first molars when they are taken out, it is not at all certain that they will do so. If the sixth year old molar is to be extracted it must be done before the twelfth year old molar makes its appearance. After it has appeared the extraction of the sixth year molar should be avoided if possible. I confess that I find the third molars frequently wanting. Again I find that two of the *dens sapientiae* will be represented and no more.

Dr. Bonwill.—On the conservation of the sixth year molar. The older I am in dentistry the more conservative I become of every tooth. And if there is any one tooth for the preservation of which I am more particular, it is the sixth year molar, for there is no one tooth the loss of which disarranges the articulation of the teeth more than the loss of this one. By all means save every tooth you can, gentleman. If you commence early enough you will not need to do crown and bridge-work.

In regard to operative dentistry we have not a system. There is nothing known of the general practice of dentistry. If a patient goes from one dentist to another he will not know what has been done by the first dentist.

Dr. Sweeney.—I would like to say a word or two. Dr. Genese said he proposed to attack the paper in its weakest point, copper amalgam. What I said about copper amalgam was not from individual experience, because my experience with it has been in letting it alone. I have used a little of it. I am not a success as a chemist but express the views of those who are. One complaint of it is not that you never find a good filling, but that you are utterly unable to determine what it is going to do. That it is utterly unreliable. He claims that it is not the black colored ones but the gray ones which turn out badly.

In regard to Dr. Timme's preparation I don't think you will find that in my paper I recommended it unduly. I wanted to bring in a few things which were new. I mentioned Dr. Timme's preparation because it was new.

Dr. Joseph Roach.—What must I do with the sixth year molars ?

This is a question which confronts every operator in his examinations of the maxillas in female patients ten years old and male patients eleven in age, and requires much thought before deciding as to the retention or extracting them, for we will certainly add or detract much from the use and preservation of the patients' teeth.

I have, during the last three years, had quite a number of children patients, and have noticed that in nine of ten cases, the sixth year molars ought to be extracted.

In nearly all American children (Caucasian) we find that there is not room in the arch for the teeth The wisdom teeth are frequently poorly developed and out of the arch; the six anterior teeth and bicuspids are frequently irregular. These, I think, are good reasons for extracting the sixth year molars. We give room to the twelfth year molars, make space for the perfect development of the wisdom teeth, and better space and preserve the teeth of the entire arches. When the patient has a weak constitution, or if the teeth are badly formed, (as to their composition in structure), they are undoubtedly improved by extracting the sixth year molars. The formative and sustaining organs, (the dental arteries, veins and nerves), are certainly made more vigorous by taking away about (5-27) five-twenty-sevenths of the teeth depending upon them.

In exhibit "Model A" the patient, male, age 17, the inferior sixth year molars were extracted December, 1890, and the superior sixth year molars were extracted December, 1891. You will notice that the spaces in the inferior maxilla made by extracting the sixth year molars have entirely closed, that the other teeth are well set in the arch, just touching on proximal cutting edges, and that there is good space in maxilla for development and eruption of the wisdom teeth. In the superior maxilla where the extractions were a year later, the spaces are about two-thirds closed, and will in time be nearly as well arranged for other teeth as in the inferior maxilla.

Exhibit Model "B": patient, female; thirteen years old next May. Her teeth were crowded and soft. The superior bicuspids have fissure decays. The right inferior second bicuspid was decayed when erupted, brought about by a febrile condition during the forming of the tooth substance. After studying the entire mouth or maxillas, I thought it best to extract the four sixth year molars, which I did last March. In the inferior maxilla the right 12th year molar is entirely erupted, the left 12th year molar is beginning to show through the gum; the bicuspids are spaced, and in a few years I expect to have a fine arrangement of the inferior teeth. The superior maxilla is much better. The bicuspids are well spaced; the right twelfth year molar is entirely out of the gum, the left twelfth year molar is just erupting and the space of the sixth year molars is entirely closed. The patient will surely have a better set of teeth than if the sixth year molars had been retained.

I am confident, that since the sixth year molars are first erupted, subject to all the influences of the changes taking place during the replacement of the temporary by the permanent teeth; and that as most arches are crowded,

2

that the sixth year molars ought to be extracted, for girls about the tenth year and for boys about the eleventh years. I submit as good evidence for their extraction exhibit model C: Patient, male; age, 25. Dr. Jack, his dentist, extracted the sixth year molars at the age of ten, the patient said, for the sole purpose of trying to give him a good and well arranged set of teeth. He also informs me that every member of his family except himself have bad teeth, and that in all the others the sixth year molars were left in the arch. You will see from the model that the teeth are well arranged, the wisdom well developed and of full service, and that they are good masticators. Dr. Jack, of Philadelphia, certainly did this gentleman a great favor by extracting his teeth, and as I said before we would confer equally as good favor on nine out of ten children by extracting (in time) their sixth year molars.

---

### BRIDGE AND CROWN-WORK.

---

BY F. W. SCHLŒNDORN, D.D.S.

---

It is not my object to speak of a special method of crown and bridge-work, but of the numerous difficulties and inconveniences which are sometimes connected with it. When we consider the comfort and relief that the modern system of bridge-work brings to the patient, it is difficult to understand the prejudice which that branch of dental art has hitherto experienced, and, to some extent, still does experience. This is partly due undoubtedly to the conservative tendency of the profession, which is properly skeptical to all innovations not sanctioned by time, and partly perhaps to the bad results which only too often have been met with.

So that from being the depised sister of our dental art, she has blossomed under the care of skillful hands and now dressed in a more modern garb, has eclipsed like the beautiful cinderella all her sisters in advance of the profession and forming the hand-maid in the art of every successful modern operator.

As I have remarked before, bridge-work has suffered considerably by opposition, and ofttimes have I heard from patients and my professional brothers that bridge-work is a failure; and which would scarcely maintain its own position in dental profession, as there are too many failures connected with it.

*Gentlemen*, whoever wishes to do bridge-work successfully, must have for his motto: A thorough knowledge of the work and a great deal of honesty!

It is an interesting question to consider where the greatest errors in bridge-work are committed. In adjusting and fitting of bands for crowns and roots, and the preparing of teeth, which are to be used for the attachment of the work, we should never, under any circumstances, do the work after a (plaster of paris) model, which I am sorry to say is sometimes done. It is a matter of impossibility to do this correctly, even if the model has been trimmed and prepared by a master-hand. The band will either go too far under the free margin of the gum, or not enough, or it is too wide, and will press too tight on the gums and these committed faults will either produce inflammation of the gum, or the ruin of a tooth, which is ofttimes the case.

Herewith I will relate a few cases, which came to me:

I will tell of a case, which came to me two years ago, and which will show you that bridge-work, when not properly done, will be a failure, after wearing it for a few weeks.

The general appearance of the mechanical work was quite nice, but when I took my excavator I found I could push my instrument about 1-16 of an inch under each cap, and found the caps entirely free of cement, which I judge was due to the improper fitting of the caps and cementing of the work. At some places the work did not reach, and on others they extended entirely over the gums.

Here, gentlemen, is a case, where patient and dentist will condemn bridge-work! The patient requested to have the work done over, as she was going to leave the city and did not feel satisfied with it. After removing the bridge-work, I found the twelfth year molar and first bicuspid in lower jaw filled with amalgam. I removed both fillings, which I always do with old fillings, to see if the cavities are in proper condition. But in these I found that they had not been properly cleaned, and decay was going on, and in a short time the teeth would have been abscessed from a dead nerve. I treated and refilled those teeth, then made proper bands and crowns to fit, and replaced the bridge-work.

Another case, which seemed to be perfectly harmless to the dentist, but which produced a great deal of trouble to the patient, came to me a few months ago. The work was done a year ago, the patient suffered with inflamed and sometimes severely swollen gum; the work was attached to both eye teeth with Dr. Evans' gold crowns. By examining the troublesome place, I found that the dentist had taken a plier and pressed the cap of a crown together to make it smaller, and this produced an elevated point under the gum, from which the irritation was produced. To overcome this trouble, I exposed the place by pressing cotton under the gum, then cutting down the point with a corrundum and then burnishing it. In this case I would call to your attention, that dentists who do bridge and crown-work should be particularly careful in burnishing and polishing the bands, which go under the free margin of the gums.

And it is true there are cases, in which the work turns out beautifully in every respect, where we are delightfully conscious that all is in good order and must necessarily crown our effort with success!

Yet after all, after a year or two, the patient returns to the dentist's office, and the dentist can generally observe by the dissatisfied look of the patient that something is wrong, which proves either that a facing has been cracked, or a crown split, or, which is still worse, the shaking of the bridge-work, on account of the roots becoming loose!

Now what to do? Now is the time for the dentist to bring his ingenuity in play, to alter the difficulty, so that his patient can leave his office with satisfactory results.

A patient came to me wearing a bridge-work on both sides of upper jaw, from the first bicuspid to twelfth year molar (inclusive). This work was one, which in all respects was perfectly and beautifully done, but yet was about to lose its durability, as the teeth upon which the work was fastened became loose! The patient still possessed her six front teeth, and to accomplish my ideas, had to sacrifice both eye teeth. The patient, a very intelligent lady, was entirely satisfied, as I explained to her that this was the only way to repair the work. To start with I removed the bridge-work, then cut off both eye teeth, prepared a cap with a cylinder, fitted over the root, then I replaced the bridge-work temporarily and united the bridge-work on cap together with wax, invested it in plaster of Paris and soldered, then cemented both parts of bridge-work. Now I swedged a cap over the other, which was soldered to the bridge-work, filed a pivot of gold iridium which was cleft, and fitted exactly into the cylinder. The cap and pivot were then soldered together and fixed upon the eye-tooth cap. Then I took the porcelain facing which was backed with gold, fastened it to the cap and pivot, and soldered both parts together. Then I set the eye-teeth into their position, took a plaster impression (of the front part of the mouth) and formed from this a zinc model, then swedged upon it a gold plate, which was soldered to both eye-teeth and combined them. This completed the work.

Another case, where the cap over a twelfth year molar of a full upper bridge-work was split on the buccal surface. To remove the work would have given a great deal of trouble, and have been very expensive to the patient. My object was to repair the work in the mouth. To start with I took an impression of the split surface, then made a zinc die from the impression and swedged a small gold plate to cover the side of the split crown. I next drilled four holes through the plate, cap and enamel of the tooth; then made four platina iridium screws, cemented the plate to the split crown and fastened it with the screws to the cap and tooth. This work has been done nearly a year and a half, and has given entire satisfaction.

A similar case is to repair a broken facing, which you all perhaps have had occasion to do, and to repair it in such a way that no one can discover it to be a repaired case. I take an impression of the backing of the broken face, then make plaster model of the impression, grind the facing to the model, so that it will fit perfectly, then cover back of facing with a piece of paper, as making a gold backing, put the paper on backing of broken face, and I find the exact place to drill the holes for the pins, then cement the facing in the place and rivet.

Gentlemen, I have now finished in regard to bridge-work, but would with your kind attention make a few remarks in regard to an artificial superior maxillary of the left side, a case which I did some eight years ago in Europe. The patient, a gentleman, about 29 years of age, lost the left side of the superior maxillary by an operation. To replace the deficiency, I had taken an impression with modeling composition and from this impression made a rubber plate to cover the roof of the mouth and extend over the lost surface of the plate. I then built up with gutta percha the lost superior maxillary to get the exact shape. I next invested this in flask, then removed the gutta percha, packed the same with rubber, and vulcanized; to overcome the heavy weight I made the case hollow. To keep this plate and work in position, I cemented a gold crown which I made square shape over the twelfth year molar in lower jaw left side, then made another crown to fit over the first one, and to this crown attached a gold spring, which fastened to the work and in this way kept it in position.

DISCUSSION.

Dr. Bonwill.—Against general bridge-work such as I usually see I am opposed. The most of what I have seen is apparently worthless. I have seen so much mutilation from this cause. It is a good thing in itself, but it has been carried too far. In the majority of cases, which I have seen, it has been the cause of irritation and deposit, and the root has gone in spite of it. I am in favor of removable bridge-work. Simply resting it on the gum and having little anchorages on the teeth. I would not object to putting a filling into a tooth and anchoring to the filling. Last year I showed you a plan of removable bridges. I am against permanent work in a majority of cases. I don't say that such work should never be done, but that a great deal of judgment should be exercised in doing it. Only a few men are capable of doing it.

Dr. B. Holly Smith.—Much injustice has been done to the so-called bridge-work by an improper selection of cases. Some have undertaken bridge-work when it should never have been done. From this has come its condemnation. Under proper circumstances bridge-work is a God-send. I question the propriety of cushioning the gum to retain particles of food and secretions. It is impossible to keep out these secretions. I never removed a saddle in my life where the gum was not inflamed and in anything else than a healthy state. I propose to criticise or oppose any appliance which has a cushion resting on the gum, I have never seen a case where it did not bring trouble. In regard to the necessity of preparing the work in the mouth my plan is not to make the bridges on the teeth themselves. I first take an impression and send it to the laboratory where I have a skilled man who makes the crown. He sends me the band and I see that it is perfectly fitted marking any corrections, which need to be made. I return it to him, and he cuts it as I direct. I don't think the band can be as well fitted on the teeth as having it done in the laboratory. I prefer to fit a collar on a cuspid tooth in the mouth. I have never seen cement which would stand in all mouths. I tell a patient in whose mouth I cement bridge-work to come to me again in three months.

I think if we are careful there is no reason for the wholesale condemnation of bridge-work.

Dr. Genese.—Tin-foil has been mentioned as useful in getting a pattern of the teeth. Tin is a brittle substance. If you take lead foil you will find it much better. If you try it, you will be surprised how nearly perfect you can make your work fit."

